# A New Approach for Infrared Temperature Measurement Sensor Systems and Temperature Control for Domestic Induction Hobs

**DOI:** 10.3390/s25010235

**Published:** 2025-01-03

**Authors:** Hakan Altuntaş, Mehmet Selçuk Arslan

**Affiliations:** 1 Central R&D Department, Beko Corporate, Tuzla, Istanbul 34950, Türkiye; 2 Mechatronics Engineering Department, Yıldız Technical University, Besiktas, Istanbul 34349, Türkiye; msarslan@yildiz.edu.tr; 3 School of Computing, Engineering and the Built Environment, Glasgow Caledonian University, Glasgow G4 0BA, UK

**Keywords:** home appliances, induction heating, induction hobs, infrared temperature sensor, non-contact temperature measurement, radiation theory, sensor systems, temperature control, thermopile

## Abstract

The accurate measurement of cooking vessel temperatures in induction hobs is crucial for ensuring optimal cooking performance and safety. To achieve this, improvements in existing measurement methods such as thermocouples, thermistors, and infrared (IR) temperature sensors are being explored. However, traditional IR sensors are sensitive to interference from the heated glass ceramic, severely affecting accuracy. This challenge is addressed by introducing a new sensor system with an optical filter designed to match the glass ceramic’s optical characteristics. The theoretical model presented here proposes the separation of the total radiation reaching the IR sensor into components emitted by the cooking vessel and the glass ceramic. However, the radiation component originating from the glass ceramic mentioned here is significantly higher than the radiation component of the cooking vessel, which creates difficulties in measuring the temperature of the cooking vessel. Simulations and real cooking experiments validate the model and demonstrate that the optic filter significantly increases the contribution of pot radiation to the sensor measurement. This causes a more accurate reflection of the actual cooking vessel temperature, leading to improved temperature control and enhanced cooking experiences in domestic induction hob appliances. This research contributes to the field by innovatively addressing challenges in real-time temperature control for induction cooking appliances. The elimination of pot dependence and improved accuracy have significant implications for cooking efficiency, safety and food quality.

## 1. Introduction

Domestic appliances have continuously evolved, transitioning from conventional gas burners to modern cooking hobs utilizing induction heating (IH) technology [1,2,3]. IH is a non-contact heating technique that relies on subjecting the target to be heated to an alternating magnetic field [4]. Moreover, IH is favored for domestic use due to its rapid heating, efficiency, precise control, safety, and cleanliness [5].

Conventional induction hobs predominantly offer two primary control methods: power control and temperature control. From the present technological landscape and literature, it is evident that many induction hobs are predominantly controlled using the power control method [6]. This entails users selecting their desired power level (e.g., 1, 2, 3, Boost) via the hob interface (display, buttons, etc.) for operation. Traditional power- control methods, while offering user flexibility, often lack the repeatability and energy efficiency required for modern culinary practices.

In contrast, temperature-controlled induction hobs, especially those employing closed-loop control with sensor feedback, have demonstrated superior performance in achieving standardized cooking outcomes and optimizing energy consumption [7]. Understanding the cooking process and quickly translating this knowledge into cooking assistance are crucial, necessitating the use of innovative techniques that preserve traditional methods, such as the implementation of temperature control [8]. Temperature-controlled induction hobs are typically categorized into two main groups: open-loop controlled and closed-loop controlled. In open-loop controlled hobs, there is no direct temperature measurement sensor system. Instead of allowing for users to select the power level, predefined auxiliary cooking programs based on timers and interface settings are provided. Conversely, closed-loop controlled hobs incorporate a sensor system to measure the temperature of the cooking vessel directly or indirectly. The hob then endeavors to maintain the cooking vessel at the temperature value specified by the user or automatic programs.

Compared to temperature-controlled hobs, power-controlled hobs grant users full control over their health and quality of cooking [9]. However, this approach often leads to challenges in replicating standardized cooking procedures and a decrease in system efficiency due to wasted energy that does not contribute to the cooking process [10]. In contrast, temperature-controlled hobs offer consistent and highly controllable cooking experiences, ensuring standardized cooking outcomes with each use. This approach also minimizes the risk of undercooking or overcooking, which can lead to foodborne illness [11]. Consequently, significant improvements in cooking quality, health benefits, and user convenience are observed. Moreover, energy efficiency is enhanced, and safety risks are minimized in such systems [12]. Table 1 highlights key performance differences and advantages of each approach.

The pursuit of precise temperature control in induction cooking has gathered significant attention in recent years, driven by the demand for healthier, more consistent, and energy-efficient cooking experiences. Monitoring the temperature of food inside cooking vessels facilitates healthier food preparation, ensures optimal cooking outcomes, and enables autonomous cooking [13]. Among various cooking platforms, induction hobs stand out as having the greatest potential for integrating these technologies.

**Figure 1:**
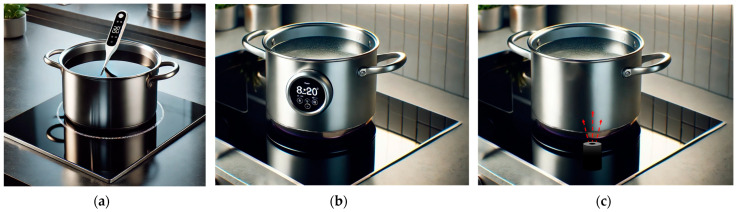
Induction hob temperature measurement methods: (**a**) temperature probe placed inside the cooking vessel; (**b**) an apparatus placed on the surface of the cooking vessel; (**c**) on-hob temperature measurement systems.

Various sensor placement strategies are encountered in temperature-controlled induction hobs, including probes placed inside the cooking vessel (Figure 1a) or on its surface (Figure 1b). While these methods offer high measurement accuracy, they often raise concerns regarding usability [14,15]. Alternatively, on-hob temperature detection with IR sensors (Figure 1c) provides a more user-friendly option but may encounter issues due to surface contamination and environmental factors.

**Figure 2:**
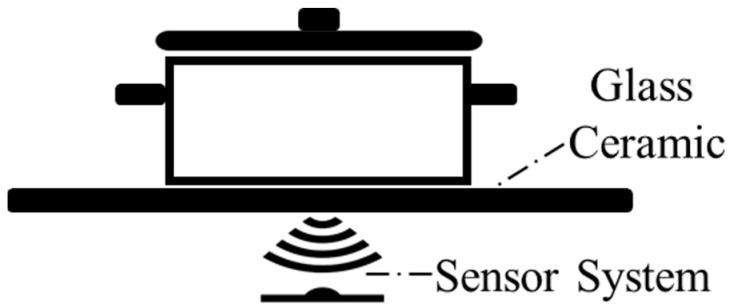
Non-contact temperature measurement inside the induction hob.

Non-contact temperature measurement systems, such as those utilizing IR sensors positioned beneath the glass ceramic, offer distinct advantages. This approach ensures seamless integration with the appliance design, minimizes user intervention, and enhances comfort [16]. By measuring temperature without direct contact, these systems avoid the challenges of physical processes while maintaining reliable performance (Figure 2).

Traditional IR temperature sensors, such as thermopiles, are commonly used for various non-contact temperature measurement applications, including automobiles, household appliances, and medical devices like clinical ear thermometers [17]. Specifically, household appliances, ranging from microwave ovens to hairdryers, provide numerous opportunities for non-contact temperature sensing [17]. Imaz et al. further explored the implementation of an IR thermometry system designed for IH, highlighting its effectiveness in providing accurate temperature readings [15]. Lasobras et al. investigated the application of IR sensor-based temperature control in domestic induction cooktops, demonstrating significant improvements in heat distribution and cooking efficiency [16].

Backherms et al. published a patent that introduces an enhancement in the accuracy of temperature detection sensors placed under glass ceramic by utilizing magnetic shielding. The patent describes key components including a sealing device for thermal isolation, a magnetic shielding device composed of ferrimagnetic or ferrite materials, and an optical shielding device for controlling thermal radiation. Two different IR sensors, an LED mechanism, and other components ensure precise temperature measurement of the cooking vessel by filtering radiation from the glass ceramic and utilizing homogenous thermal distribution methods. In this patent, the IR sensor’s window materials are customized for reading glass ceramic (high-pass, from 5500 nm) and cooking vessel (band-pass, 3100 nm–4200 nm) [18].

Another patent describes an induction cooking device designed to monitor the temperature gradient or absolute temperature using an IR sensor for detecting IR radiation from the cooking vessel and a contact temperature sensor, which measures the cooking vessel’s temperature through thermal conduction. The patent focuses on the system ensuring safety by cross-verifying sensor readings, using the contact sensor as a fallback if the IR sensor malfunctions [19].

An induction cooking system that integrates a temperature sensor, positioned beneath the glass ceramic, specifically an IR temperature sensor, to detect IR radiation emitted from the heated object is described in [20]. There are two elements of the sensor system: one of them is used to characterize the cooking vessel’s surface with help of the table for compensation of the temperature measured by IR sensor and the second one is the IR temperature sensor. The patent also mentions the use of a filter to block radiation from the glass ceramic surface of the induction hob, which the patent refers to as the upper plate; however, it does not provide any specific details or specifications regarding the filter [20].

The difficulty of accurately measuring low temperatures (70–150 °C) with traditional glass ceramic cooking surfaces due to their low transmittance is addressed in [21]. The patent proposes new glass ceramics with higher transmittance (2900–4200 nm) and suggests an IR sensor with spectral sensitivity between 2800 nm and 4400 nm, ideally 3600 nm, located beneath the glass ceramic. A thinner glass ceramic reduces IR absorption, improving measurement accuracy. The patent also recommends user input for pot emissivity and suggests placing the sensor below the heating coils, shielded by a guide tube. Tests showed that the proposed solution reduces temperature overshoots by 15% and speeds up heating compared to standard glass ceramics [21].

Carretero et al. presented a radiation heat measurement model for temperature estimation in IH appliances, exhibiting the potential for precise temperature control [22]. However, the accuracy and precision of IR sensors, located beneath the glass ceramic, are significantly compromised due to interference from the heated glass ceramic itself, which emits thermal radiation that affects the sensor’s measurement of the actual cooking vessel temperature [23]. This leads to inaccurate measurements and challenges in maintaining consistent cooking temperatures [14].

To address this limitation, this paper presents a new sensor system designed to achieve highly accurate cooking vessel temperature measurement on induction hobs. The proposed system incorporates an innovative optical filter specifically chosen to address the optical characteristics of the glass ceramic. We explore the theoretical framework behind the sensor system, including a detailed model that separates the total radiation reaching the IR sensor into its constituent components: radiation from the pot bottom, the glass ceramic’s emission, and reflections and transmissions within the system.

This research employs a combination of simulations and real cooking experiments to validate the effectiveness of the proposed sensor system. The simulations model the sensor response with and without the optical filter, while the experiments involve measuring temperatures of the pot, glass ceramic, and sensor system under controlled conditions. The results demonstrate that the optical filter significantly improves the contribution of cooking vessel radiation to the overall sensor measurement, leading to a more accurate reflection of the actual cooking vessel temperature. It is shown that the proposed sensor system successfully overcomes glass ceramic interference, enabling clearer measurement of cooking pot temperature. Experimental results, supported by the computer simulations, demonstrate the model’s accuracy and effectiveness.

## 2. Proposed Sensor System and Sensor Selection

**Figure 3:**
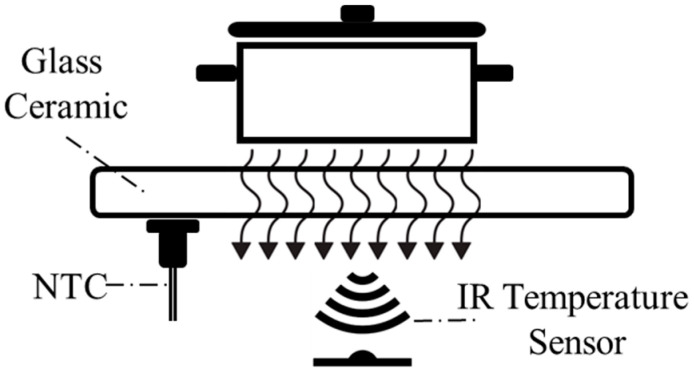
Proposed sensor system for temperature measurement.

Direct measurement of the cooking vessel’s temperature using an IR temperature sensor is not possible due to the complexity of total radiation reaching the IR sensor. Interference from the heated glass ceramic plays a crucial role in that complexity. For those reasons, understanding the glass ceramic temperature is crucial for accurately analyzing the radiation components that contribute to the total thermal signal reaching the sensor. To achieve this, a separated sensor is employed to directly measure the temperature of the glass ceramic [23]. In this case, a negative temperature coefficient thermistor (NTC) sensor is utilized in direct contact with the glass ceramic (Figure 3).

### 2.1. Infrared Temperature Sensor Selection

For non-contact temperature measurement of the cooking vessel, an IR temperature sensor was selected. IR temperature sensors convert thermal radiation into electrical signals, allowing for accurate temperature measurement without physical contact [24]. Also, over the past decade, IR temperature sensors have gained growing attention due to their dependable performance and outstanding cost-effectiveness [25].

In selecting the appropriate IR temperature sensor for non-contact measurement in induction hobs, several parameters were meticulously considered:

- Measurement accuracy;
- Electrical noise resistance;
- IR measurement wavelength range;
- Measurement range;
- Operating temperature range;
- Field of view (FoV).

In addition to the selection parameters mentioned above, when factors such as the cooking vessel’s uncertain emissivity, the glass ceramic’s temperature, environmental conditions, and radiation significantly affect measurements, it becomes essential for the sensitive IR sensor to deliver durable and repeatable results. Also, the high electrical noise, fluctuating environmental temperatures, and harsh conditions in induction hobs make analog sensors difficult to use. Proper isolation and careful calibration of analog sensors are critical. To address these challenges and ensure reliable performance at even low temperatures, this system design exclusively utilizes digital sensors. Digital IR sensors are also known for their fast response times, high accuracy, and ease of integration into digital systems.

**Figure 4:**
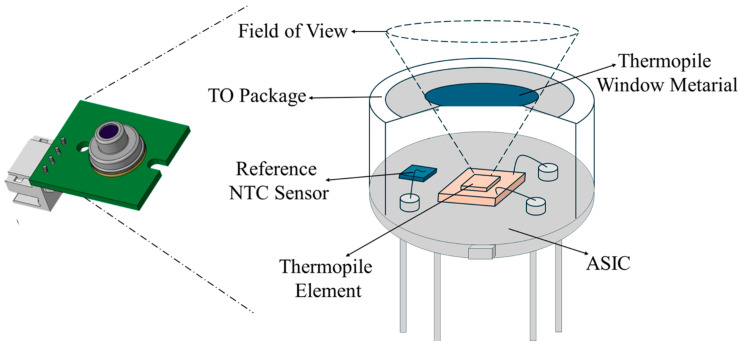
Detailed cross view of used digital IR temperature sensor.

As shown in the Figure 4 schematic, the working principle of a digital IR temperature sensor (thermopile) relies on detecting the IR radiation emitted by an object. The IR sensor includes a window material, thermopile element, reference NTC sensor, and application-specific integrated circuit (ASIC). The window material determines sensor’s wavelength range (transmittance), defines the FoV in conjunction with the thermopile element, and contributes to sealing. The thermopile element acts as the sensing component, converting absorbed radiation into a voltage signal. Here, thermopiles operate based on the Seebeck effect, which has long been utilized in conventional thermocouples [26]. The reference NTC sensor measures the temperature of the sensor package, specifically the temperature of the cold junctions, to calibrate thermopile element measurement. All measurement signals are then processed and converted into a temperature reading with the help of the ASIC. The sensor then communicates with the system via the digital system management bus (SMBUS) communication protocol to transmit temperature values.

#### 2.1.1. Experimental Studies for IR Temperature Sensor Performance

**Figure 5:**
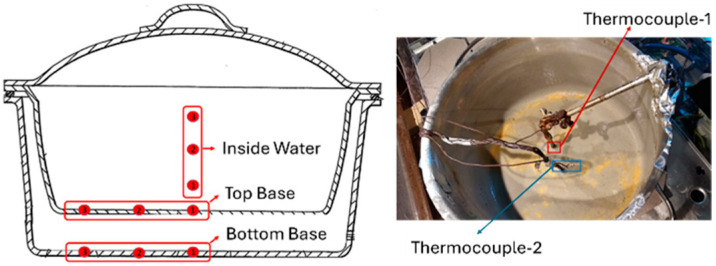
Reference temperature points on the cooking vessel.

Based on these criteria, four different IR temperature sensors were identified as potential candidates. These sensors were initially tested using the same window material, which had a transmittance value of 80% ($\tau_{w}=0.8$) across the entire spectrum. Additionally, a slot was drilled into the glass ceramic in front of the sensor to minimize the impact of the transmittance parameter and the radiation emitted by the heated glass ceramic on the sensor’s performance. According to this instruction, an experimental test setup was established to select the most ideal IR temperature sensor. In this test setup, sensors were placed in the induction hob and real-time cooking tests were performed. Throughout the tests, temperatures were recorded using reference thermocouple temperature sensors from various positions on the cooking pot (Figure 5). This approach facilitated the assessment of the sensors’ variability and measurement performance relative to the reference. Additionally, the emissivity values of the tested stainless-steel pots were approximately 0.29 (Pot A) and 0.86 (Pot B).

The experiments were designed using the design of experiment (DoE) methodology via Minitab statistical software (v21.1.0) [27]. The DoE study methodically examines the effect of input variables (factors) on the sensor output variable (response) to clarify the performance of the sensors. Table 2 presents the experimental design parameters used to evaluate the performance of different IR temperature sensors to systematically assess how various factors, such as cooking vessel material, power levels, and temperature ranges, influence sensor performance. Each experiment was performed with a specific sensor, cooking vessel, power level, and temperature range to analyze the accuracy and reliability of the sensor’s readings. This approach allows for a detailed understanding of sensor behavior under real-world cooking conditions, ensuring the suitability of the selected sensor for induction hob applications.

**Figure 6:**
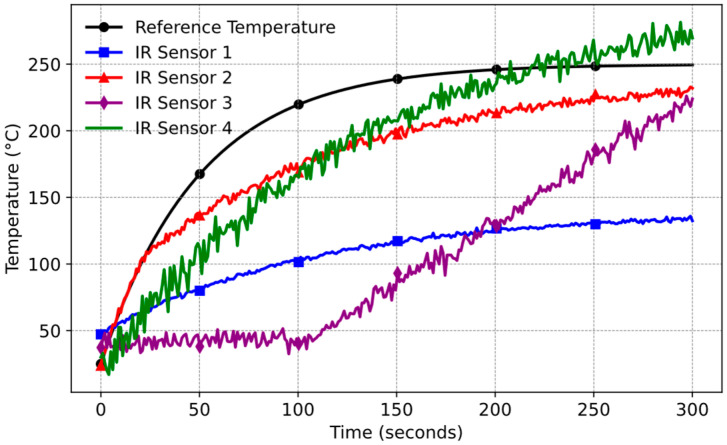
IR temperature sensor measurement results (Pot B and ε = 0.86).

Throughout these experiments, reference temperature data were collected via a high-precision Agilent 34950A Datalogger device [28]. Electrical parameters such as power, energy, current, voltage, and frequency within the system were recorded using the Chroma Power Analyzer device [29]. The IR sensors were tested under controlled conditions, with their responses recorded and analyzed over the same period (Figure 6).

Based on the experimental results, IR Sensor 2 is suitable for its balanced performance. It provides a wide measurement range, accurately follows the reference signal initially, and maintains an acceptable noise level.

#### 2.1.2. Selection of Window Material for IR Temperature Sensor

**Figure 7:**
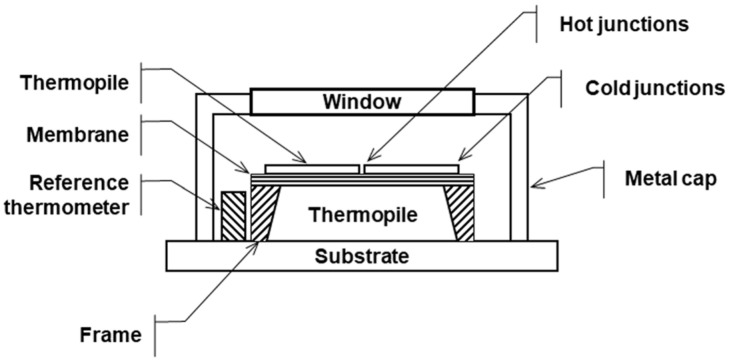
Structure of the IR-based temperature sensor [30].

The window material of thermopile allows for specific IR wavelengths to pass through while blocking unwanted wavelengths and interferences. As shown in Figure 7, the thermopile-based IR temperature sensor consists of several key components: the window, thermopile, membrane, reference thermometer, and a protective metal cap. The window plays a critical role by selectively transmitting IR radiation in the desired wavelength range (e.g., 3–5 microns) to the thermopile while filtering out other wavelengths that may cause measurement inaccuracies.

Since the IR temperature sensor is placed under the glass ceramic, the transmittance wavelength range of the glass ceramic plays an important role in determining the most suitable wavelength transmittance for window material of IR temperature sensor.

**Figure 8:**
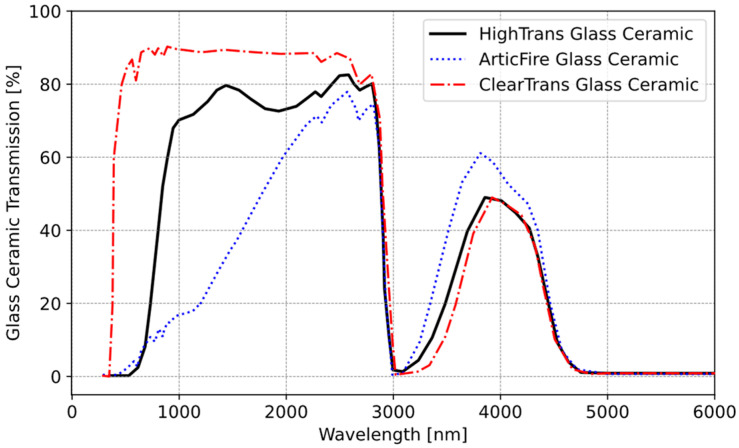
Transmission values of Schott glass ceramic.

There are three types of glass ceramics mostly used in the industry. In Figure 8, transmission information for these three glass ceramics is shown.

**Figure 9:**
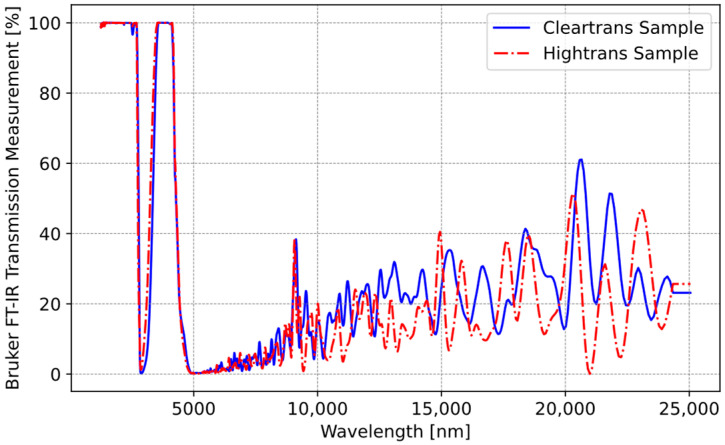
Transmission validation results of Schott glass ceramics.

In this study, our focus was on ClearTrans and HighTrans glass ceramics. These two glass ceramics were further analyzed under controlled laboratory conditions using a Bruker Fourier transform infrared spectroscopy (FT-IR) [31] transmittance measurement device and subsequently re-measured to validate their accuracy. In this device, the transmission values of the glass were measured across the spectrum up to 25000 nm. Figure 9 shows that the datasheet values were validated.

The validation of the glass ceramic transmission study also shows that the IR transmittance of the glass ceramic gradually ends at approximately five micrometers. Following this stage of the work, HighTrans glass ceramic was used.

**Figure 10:**
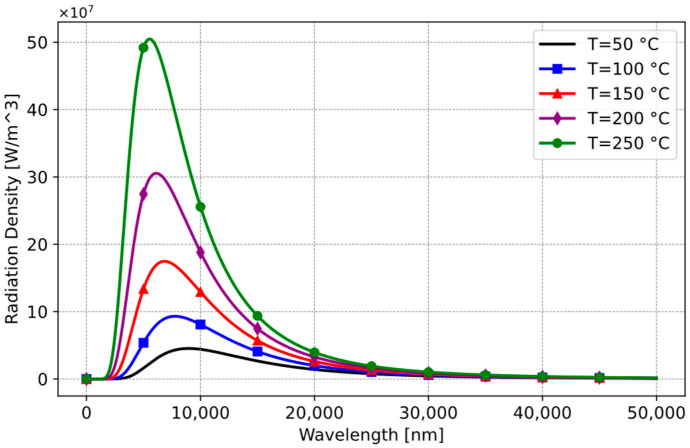
Planck’s law on black-body radiation.

Another variable influenced by wavelength is the actual temperature of the cooking vessel. Thermal radiation emitted from a cooking vessel fluctuates across different wavelengths based on its temperature (Figure 10).

**Figure 11:**
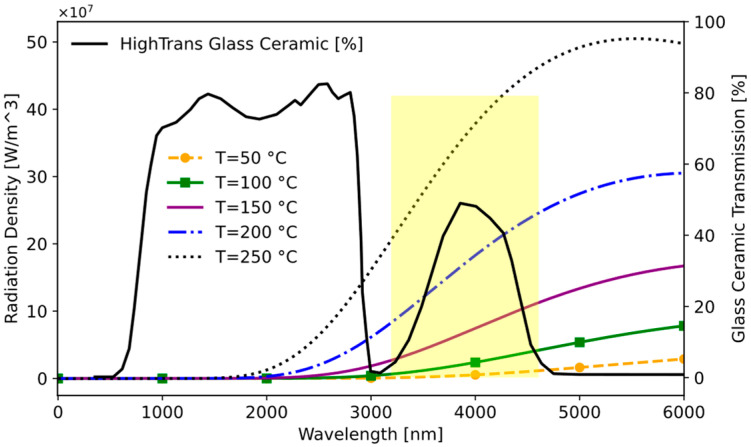
Operational wavelength range for induction hob.

When thermal radiation from various temperatures is layered over the region, where the transparency of the glass ceramic is noticeable, the operational range becomes clear, as demonstrated in Figure 11.

**Figure 12:**
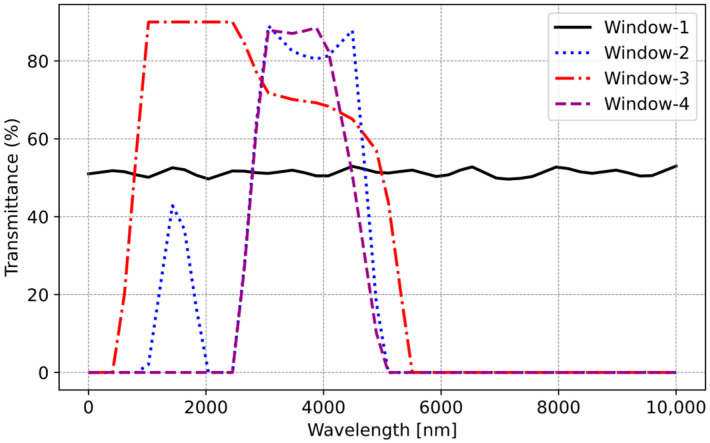
Transmittance of IR sensors.

In the cooking process, temperatures typically range from 50 °C to 240 °C. Analysis of this temperature spectrum in Figure 11 reveals a noticeable increase in energy emission beyond approximately 3000 nm. Beyond this threshold, the glass ceramic’s transmittance imposes limitations on the measurement. In summary, the sensor effectively captures wavelengths within the range of 3000–5000 nm. Consequently, it is optimal for the selected sensor to operate within this wavelength band. There are four types of window material (Window-1: uncoated silicon; Window-2: coated silicon band-pass filter (BPF); Window-3: germanium; Window-4: zinc sulfide) for selected IR Sensor 2. These window materials determine the transmittance level of the IR sensor. Utilizing this information, the transmittances of each sensor are presented in Figure 12. Window-2 and Window-4 exhibit the highest transmittance percentages within this range, making them usable for our application.

Another factor to consider is the operating temperature and measurement range of the sensor. The ambient temperatures within the existing induction cooktop platform, where the sensor will be situated, must withstand at least 75 °C according to Underwriters Laboratories (UL) and Verband der Elektrotechnik (VDE) standards [32,33]. Also, when evaluating the existing cooking programs, it is determined that the highest temperature requiring measurement is 240 °C. Furthermore, upon examining the FoV angles of the sensors, it is evident that the lowest field of view angle should be selected. This narrow FoV angle thus enables the sensor’s placement at the structurally and geometrically farthest point. Therefore, detailed specification parameters of the selected sensor, considering all these factors, are provided in Table 3.

## 3. Thermal Radiation Modelling and Improvement of Sensor

### 3.1. Thermal Radiation Modelling of Infrared Emissions for Induction Hobs

**Figure 13:**
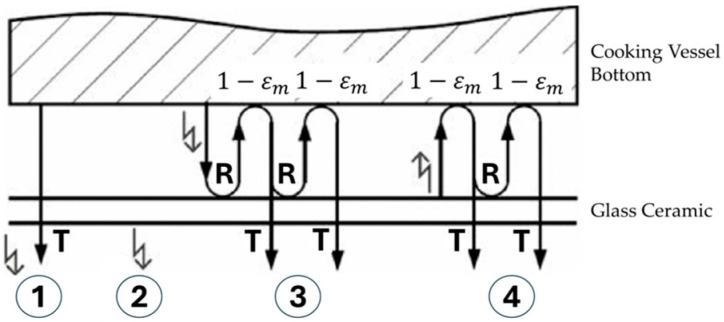
Radiation components of induction hob [22].

In induction hobs, as the cooking process begins, the cooking vessel heats up directly, subsequently heating the glass ceramic indirectly. As a result, the heated glass ceramic begins emitting thermal radiation. For that reason, the radiation detected by the sensor encompasses not only the cooking vessel’s temperature but also emissions from glass ceramic and other emissions. Without accurately modelling the total radiation reaching the IR sensor and isolating each component, precise measurement of the cooking vessel’s temperature is unpredictable. In other words, the IR sensor’s response does not solely represent the cooking vessel’s temperature. In our application, four components contribute to the total thermal radiation under normal conditions (Figure 13) [22].

Carretero et al. explained and formulated these components as follows: The first component consists of rays that can directly reach the sensor from the cooking vessel. The second component comprises the IR signal that directly reaches the sensor from the heated glass ceramic. The remaining two components involve signals that indirectly reach the sensor due to dual reflection between the glass ceramic and the cooking vessel. The third signal reaches the sensor after initially reflected from the cooking vessel to the glass ceramic, then again from the glass ceramic to the cooking vessel, and finally to the sensor. Similarly, the fourth signal reaches the sensor from the heated ceramic glass to the cooking vessel, then reflects again from the ceramic glass to the cooking vessel, and finally to the sensor. These four components are mathematically expressed by Planck’s law [34] using Equation (1), which describes the spectral radiance $i_{\lambda_{b}}\left(\lambda ,T\right)$ of electromagnetic radiation emitted by a black-body in thermal equilibrium at a given temperature T. Thus, it represents the amount of energy radiated per unit.

We can compute all thermal radiation reaching the sensor as four distinct components, respectively [22]:

(1) $$i_{\lambda_{b}}\left(\lambda ,T\right)=\frac{2hc_{0}^{2}}{\lambda^{5}}\frac{1}{e^{hc_{0}/(k_{b}T\lambda )}-1}$$

(2) $$i_{1}=\int_{0}^{\infty }\tau_{C,\lambda }\cdot \varepsilon_{P,\lambda }\cdot i_{\lambda_{b}}\left(\lambda ,T_{P}\right)d\lambda$$

(3) $$i_{2}=\int_{0}^{\infty }\varepsilon_{C,\lambda }\cdot i_{\lambda_{b}}\left(\lambda ,T_{C}\right)d\lambda$$

(4) $$i_{3}=\sum_{n=1}^{\infty }\int_{0}^{\infty }\rho_{P,\lambda }^{n}\cdot \rho_{C,\lambda }^{n}\cdot \tau_{C,\lambda }\cdot \varepsilon_{P,\lambda }\cdot i_{\lambda_{b}}\left(\lambda ,T_{P}\right)d\lambda \;\;=\int_{0}^{\infty }\frac{\rho_{P,\lambda }\cdot \rho_{C,\lambda }}{1-\rho_{P,\lambda }\cdot \rho_{C,\lambda }}\cdot \tau_{C,\lambda }\cdot \varepsilon_{P,\lambda }\cdot i_{\lambda_{b}}\left(\lambda ,T_{P}\right)d\lambda \;$$

(5) $$i_{4}=\sum_{n=1}^{\infty }\int_{0}^{\infty }\rho_{P,\lambda }^{n}\cdot \rho_{C,\lambda }^{n-1}\cdot \tau_{C,\lambda }\cdot \varepsilon_{P,\lambda }\cdot i_{\lambda_{b}}\left(\lambda ,T_{C}\right)\;\;=\int_{0}^{\infty }\frac{\rho_{P,\lambda }}{\left(1-\rho_{P,\lambda }\cdot \rho_{C,\lambda }\right)}\cdot \tau_{C,\lambda }\cdot \varepsilon_{C,\lambda }\cdot i_{\lambda_{b}}\left(\lambda ,T_{C}\right)d\lambda$$

where $T_{P}$ is the cooking vessel temperature in Kelvin; $T_{c}$ is the glass ceramic temperature in Kelvin; $\tau_{c}$ is the glass ceramic transmittance; $\rho_{P}$ is the cooking vessel reflectance; $\rho_{c}$ is the glass ceramic reflectance; $\varepsilon_{P}$ is the cooking vessel emissivity; and $\varepsilon_{c}$ is the glass ceramic emissivity. Therefore, the total signal reaching the sensor is expressed as follows in (6):

(6) $$i_{T}=i_{1}+i_{2}+i_{3}+i_{4}$$

The temperature conversion of these four components reaching the IR temperature sensor is expressed as follows (7):

(7) $$T_{Sensor}=[k{\frac{\left(i_{T}*\pi \right)}{{(\varepsilon }_{Sensor}*\sigma )\;}}^{1/4\;}]-273.15$$

where σ represents the Stefan–Boltzmann constant (5.670 × 10^−8^ Wm^−2^ K^−4^) and *k* is the calibration coefficient of the system, and it should be determined for each unique system via experimental studies.

### 3.2. Computer-Based Simulation for the Proposed Sensor System

The equations discussed in the previous section (1)–(7), which include thermal radiation components, were modeled in Visual Studio Code Environment [35] with Python programming language [36]. This model takes the cooking vessel temperature and glass ceramic temperature as input parameters, and based on these inputs, it calculates the thermal radiation components of the system and IR temperature sensor values individually.

In this context, the model was run using data simulating a cooking vessel temperature increase from 50 °C to 250 °C, in increments of 1 °C ($T_{P}=50\;{}^{\circ}C\;to\;250\;{}^{\circ}C$). Additionally, glass ceramic temperature $(T_{c}$) values were generated as a function of the cooking vessel temperature for this simulation.

In [15], the temperature of the cooking vessel was calculated by assuming a temperature difference of 20 °C between the cooking vessel temperature and the glass ceramic temperature, i.e., $T_{c}$ = $T_{P}$ − 20 °C, which is consistent with average values observed in domestic induction hobs. However, this approach is insufficient to fully capture the dynamics of glass ceramic heating. For this reason, in this work, we derived a function using MATLAB’s System Identification Toolbox [37] based on time series data collected from several real cooking tests. We developed a dynamic system model to describe the relationship between the cooking vessel and the glass ceramic. The function forecasts the glass ceramic temperature based on the cooking vessel temperature.

In addition to all these, Table 4 shows that the following parameter set was used during the simulation:

Note that during the simulation, the transmittance values of HighTrans glass ceramic ($\tau_{C}$) were used as a function of wavelength data from Figure 8. Also, emissivity values were used as ($0.95-\tau_{C}$). In other words, rather than using a fixed value, actual wavelength-dependent data were used.

**Figure 14:**
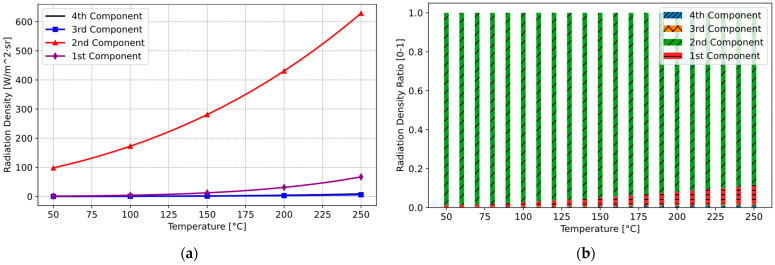
Thermal radiation and their components without optical filter: (**a**) radiation component density of the system; (**b**) proportional distribution of radiation components.

Modelling and simulating thermal radiation and their components in the induction hob across a temperature range of 50 °C to 250 °C reveals that most of the thermal radiation reaching the sensor is emitted from the glass ceramic. In addition, especially up to 120 °C, the differences between the components appear quite small. However, beyond 120 °C, there is a noticeable increase in clarity. The result of the simulation is illustrated in Figure 14. As evident from the Figure 14a, a significant proportion of the sensor measurement correlates with the temperature of the glass ceramic, representing the second component. In contrast, the first component, associated with the cooking vessel, contributes relatively little to the overall measurement. The proportional distribution of these components is depicted in the Figure 14a. Here, the ratio of the first (Red) and second (Green) components is presented, i.e., $i_{1}/i_{2}=0.036$.

These findings indicate that most of the thermal radiation reaching the sensor originates from the glass ceramic, impeding our ability to accurately measure the cooking vessel’s temperature. Accordingly, the system’s ability to make accurate measurements even at low temperatures is severely limited; it is almost impossible. To address this challenge, it is crucial to examine the relationship between optical coefficients. This relationship reveals that where the transmittance coefficient is high, the emissivity coefficient will be correspondingly low.

At this point, as discussed before, the thermal radiation emitted from the bottom of the cooking vessel reaches the IR sensor through the glass ceramic top. Therefore, the optical characteristics of the glass ceramic must be considered to accurately measure the cooking vessel’s temperature. Thermal radiation can encounter three possible scenarios: it can be transmitted through the glass ceramic, absorbed by the glass ceramic, or reflected by the glass ceramic [38]. These outcomes depend on the material’s properties transmittance (τ), absorptance (α), and reflectance (ρ). These parameters are interrelated, as expressed in Equation (8), and vary with wavelength [38].

(8) τ+α+ρ=1

Moreover, the glass ceramic itself also emits thermal radiation, which depends on its emittance (ε) parameter, similar to the cooking vessel. According to Kirchhoff’s law, the emittance of a material is equal to its absorptance (α) when in thermal equilibrium [39]. Zhang et al. assert that Kirchhoff’s law holds for semi-transparent films, provided that the Helmholtz reciprocity condition is met, even if the object is not in thermal equilibrium. Therefore, Equation (8) can be reformulated as Equation (9) [39].

(9) τ+ε+ρ=1

In our case, the glass ceramic’s transmittance is high within a specific narrow wavelength range (3–5 μm), impacting the accuracy of the cooking vessel’s temperature measurement. To resolve this issue, Backherms et al. recommend using a band-pass window material customized for the IR sensor, as described in patent EP2775784A1 [18]. This band-pass window material allows for high transmittance only within that narrow wavelength range. Consequently, the total thermal radiation reaching the sensor is influenced by the radiation emitted by both the cooking vessel and the glass ceramic. However, according to Equation (9), as the transmittance increases, the emittance decreases. Therefore, this paper suggests using an additional optical filter that increases transmittance as the emittance of the glass ceramic decreases. This filter selectively transmits thermal radiation from the cooking vessel while blocking wavelengths where the glass ceramic emits less radiation. Simulations for an optical filter with these transmittance properties show that it should identically match the optical characteristics of the glass ceramic used.

The simulations conducted to evaluate the optical filter involved modeling the transmittance properties of both the optical filter and the glass ceramic. Using computational tools in Visual Studio Code Environment [35] with Python programming language [36], we analyzed how different transmittance profiles of the filter influenced the separation of radiation components reaching the sensor. The goal was to ensure that the filter’s transmittance maximized the contribution of cooking vessel radiation while minimizing interference from the glass ceramic. These simulations were based on Equations (1)–(7) and incorporated wavelength-dependent optical parameters such as emissivity, reflectance, and transmittance derived from experimental data. The results demonstrated that an optical filter with a transmittance profile tailored to the glass ceramic’s optical properties significantly improved the accuracy of temperature measurements. For this purpose, a down-scaled optical filter with the same optical properties as the ceramic glass was manufactured.

**Figure 15:**
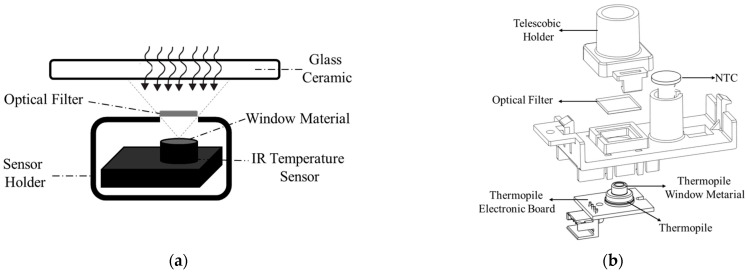
Proposed sensor system with optical filter: (**a**) schematic of the complete system; (**b**) detailed view of the sensor system.

By applying this principle and utilizing an optical filter with a transmittance coefficient matching that of the glass ceramic in front of our sensor, we can effectively minimize the influence of thermal radiation emitted by the glass ceramic on our measurement. Figure 15 illustrates the proposed sensor system equipped with an optical filter.

**Figure 16:**
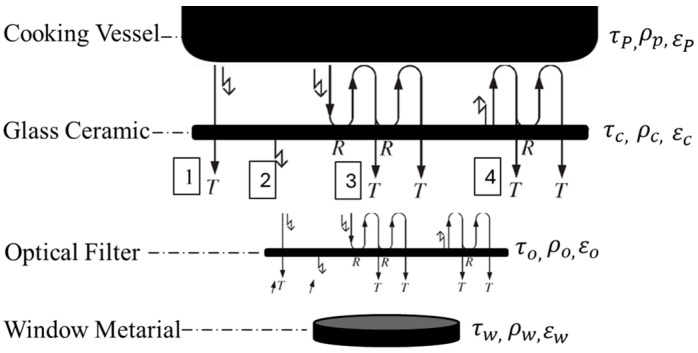
Radiation components of the proposed sensor system.

The optical filter is primarily composed of silica (SiO_2_), which is known for its excellent thermal stability, transparency in the infrared spectrum, and durability under high-temperature conditions. The silica used in this filter is of high purity to ensure minimal attenuation of the IR signal and to maintain its optical properties over extended use. Also, it is manufactured with precise dimensions to ensure compatibility with the system. Its size is 10.0 ± 0.1 mm × 10.0 ± 0.1 mm and the thickness is 0.5 ± 0.05 mm. These dimensions are optimized for integration within the infrared sensor system without affecting performance. The optical properties of the sensor’s window material or optical filter are not included in the model described earlier and expressed by Equations (1)–(7). Figure 16 illustrates the radiation components of the proposed sensor system.

According to the proposed system, the model equations are as follows:

(10) $$i_{1}=\int_{0}^{\infty }\tau_{o,\lambda }\cdot \tau_{w,\lambda }\cdot \tau_{C,\lambda }\cdot \varepsilon_{P,\lambda }\cdot i_{\lambda_{b}}\left(\lambda ,T_{P}\right)d\lambda$$

(11) $$i_{2}=\int_{0}^{\infty }\tau_{o,\lambda }\cdot \tau_{w,\lambda }\cdot \varepsilon_{C,\lambda }\cdot i_{\lambda_{b}}\left(\lambda ,T_{C}\right)d\lambda$$

(12) $$i_{3}=\sum_{n=1}^{\infty }\int_{0}^{\infty }\tau_{o,\lambda }\cdot \tau_{w,\lambda }\cdot \rho_{P,\lambda }^{n}\cdot \rho_{C,\lambda }^{n}\cdot \tau_{C,\lambda }\cdot \varepsilon_{P,\lambda }\cdot i_{\lambda_{b}}\left(\lambda ,T_{P}\right)d\lambda \;\;=\int_{0}^{\infty }\frac{\rho_{P,\lambda }\cdot \rho_{C,\lambda }}{1-\rho_{P,\lambda }\cdot \rho_{C,\lambda }}\cdot \tau_{o,\lambda }\cdot \tau_{w,\lambda }\cdot \tau_{C,\lambda }\cdot \varepsilon_{P,\lambda }\cdot i_{\lambda_{b}}\left(\lambda ,T_{P}\right)d\lambda$$

(13) $$i_{4}=\sum_{n=1}^{\infty }\int_{0}^{\infty }\tau_{o,\lambda }\cdot \tau_{w,\lambda }\cdot \rho_{P,\lambda }^{n}\cdot \rho_{C,\lambda }^{n-1}\cdot \tau_{C,\lambda }\cdot \varepsilon_{P,\lambda }\cdot i_{\lambda_{b}}\left(\lambda ,T_{C}\right)\;\;=\int_{0}^{\infty }\frac{\rho_{P,\lambda }}{\left(1-\rho_{P,\lambda }\cdot \rho_{C,\lambda }\right)}\cdot \tau_{o,\lambda }\cdot \tau_{w,\lambda }\cdot \tau_{C,\lambda }\cdot \varepsilon_{C,\lambda }\cdot i_{\lambda_{b}}\left(\lambda ,T_{C}\right)d\lambda$$

where $\tau_{o}$ is the optical filter transmittance; $\rho_{o}$ is the optical filter reflectance; $\varepsilon_{o}$ is the optical filter emissivity; $\tau_{w}$ is the window material transmittance; $\rho_{w}$ is the window material reflectance; $\varepsilon_{w}$ is the window material emissivity.

Upon implementing this solution, the components reaching the sensor were recalculated with a new simulation using Table 5 parameters.

**Figure 17:**
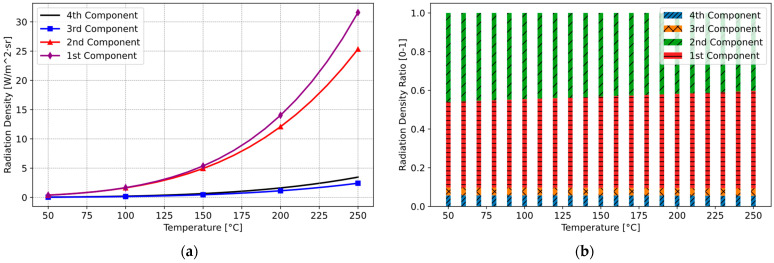
Thermal radiation and their components with optical filter: (**a**) radiation component system density; (**b**) proportional distribution of radiation components.

Following the integration of the new optical filter, the measurements of the first and second components overlap more, and the calculation results are shown in Figure 17a. Carretero et al. also presented the output signals of their IR sensor based on the radiation components for two different pot emissivity values within a temperature range of between 120 °C and 200 °C. In addition, we demonstrated that the optical filter design used in our system significantly changes the relative contributions of the radiation components, as shown in the transition from Figure 14 (without optical filter) to Figure 17a (with optical filter), covering a broader temperature range of 20 °C to 250 °C. This approach demonstrated that measurement and control can be effectively performed below 120 °C. Consequently, the radiation contribution of the cooking vessel to the overall radiation increases. Figure 17b distinctly depicts this proportional shift. In the revised system, the ratio of the first and second components is $i_{1}/i_{2}=1.094$.

It has been demonstrated that high-precision temperature measurement and control can be achieved even at low temperatures using an additional optical filter manufactured with the same optical properties as the glass ceramic. Furthermore, the additional optical filter was tested with standard thermopiles (low-cost silicon thermopiles with constant transmittance across all wavelengths, such as non-contact thermometers, as shown in Figure 12), similar to Window-1, with an 80% transparent window material. In this case, temperatures above 120 °C can be measured without the need for a customized BPF window material for the thermopile.

### 3.3. Model Validation of Simulation with Real Cooking Data

We developed a computer-based model to simulate the measurement process both with and without the optical filter in front of the sensor. To validate the accuracy of this model, we used real cooking experiment data as input. The dataset utilized in the simulation was first fed into the model without the optical filter, and then into the model with the optical filter, enabling a comparative evaluation of the outcomes.

**Figure 18:**
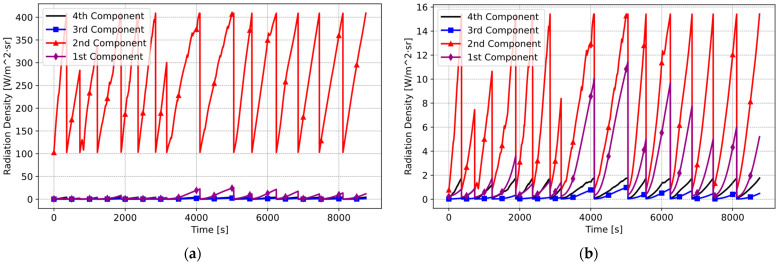
Simulation with real cooking data: (**a**) without optical filter model (Pot A and ε  = 0.29); (**b**) with optical filter model (Pot A and *ε* = 0.29).

To verify the accuracy of the results, real cooking test data from 15 distinct pan fry cooking experiments were used. The graph below illustrates four distinct radiation components plotted individually for the model without the optical filter (Figure 18a). Subsequently, an identical dataset was applied to the model incorporating the optical filter. This facilitated the recalculation of the individual radiation components reaching the IR sensor (Figure 18b).

The studies utilizing real cooking experiment data revealed that the cooking pot’s influence became significant after applying the filter.

### 3.4. Experimental Validation Results

**Figure 19:**
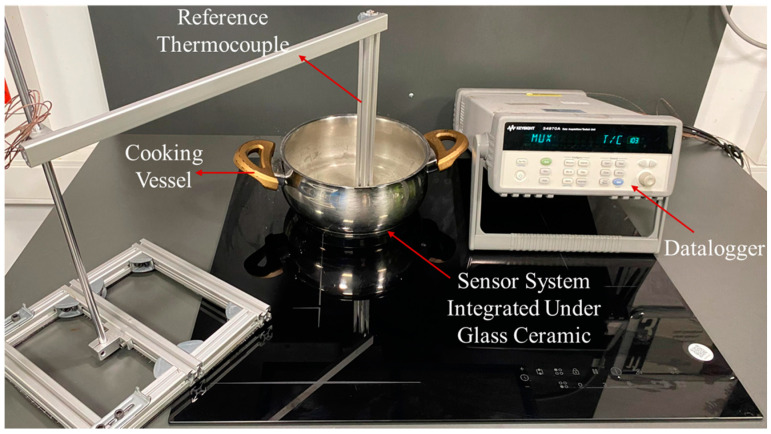
Experimental setup for model validation.

To validate the model’s accuracy experimentally, we compared the calculated values with reference measurements using a specifically designed experimental setup. In the experimental studies, the Agilent 34970A model data acquisition device [28] was used. In the shown setup in Figure 19, reference temperatures were recorded using a data acquisition system from various points including the cooking vessel, glass ceramic, ambient environment, and the sensor system.

**Figure 20:**
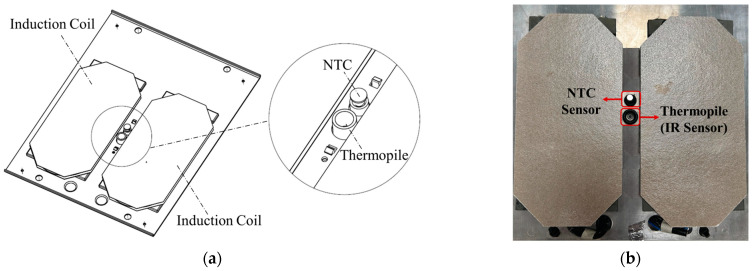
Sensor system location and assembly: (**a**) sensor system schematics; (**b**) real assembly on the induction hob.

The sensor system (Figure 15) is located within the induction hob, specifically at the center of the induction coil (Figure 20a,b).

**Figure 21:**
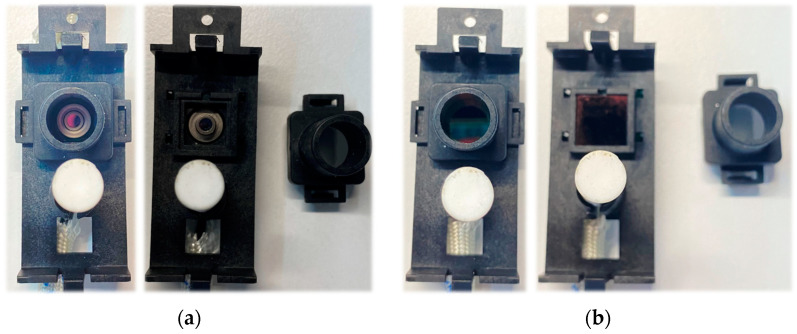
Sensor system: (**a**) without optical filter; (**b**) sensor system with optical filter installed.

The sensor system positioned between the coils includes a removable telescopic structure designed to accommodate the optical filter. In the initial experimental studies, the filter was not used (Figure 21a). Subsequently, the optical filter was inserted, and the experiments were repeated (Figure 21b).

**Figure 22:**
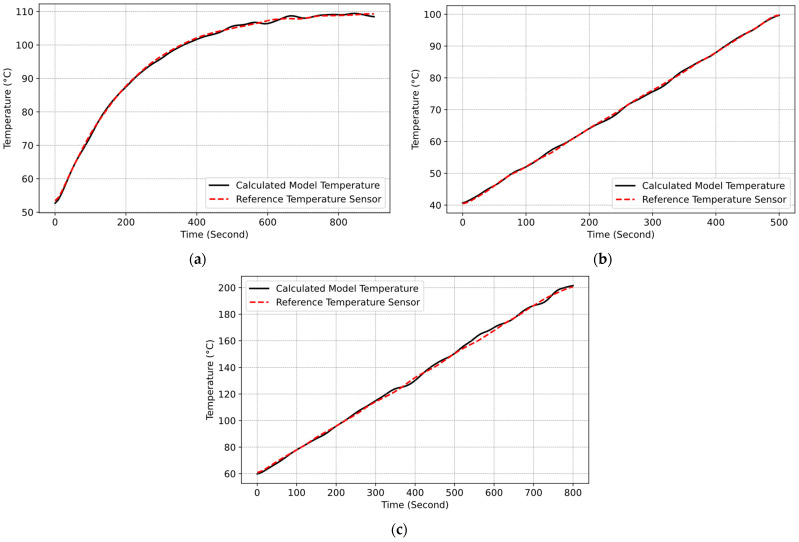
Cooking test results: (**a**) Test-1 and RMSE: 1.183 (Pot C and ε  = 0.59); (**b**) Test-2 and RMSE: 1.074 (Pot A and ε = 0.29); (**c**) Test-3 and RMSE: 1.315 (Pot B and ε = 0.86).

The accuracy of the developed model was evaluated using the recorded data of the cooking vessel and glass ceramic temperatures to compute radiation components ($i_{1},i_{2},\;i_{3},i_{4}$). These values were subsequently converted into the expected IR sensor temperature according to the developed model. The calculated temperature value was then compared with the reference thermopile sensor. To facilitate this assessment, real cooking experiments were carried out under diverse scenarios and measurements errors calculated with the root mean square error (RMSE) method. The outcomes of these cooking experiments are delineated in Figure 22a–c.

The experiment results in Figure 22a–c showed that the model accurately calculates the components of total radiation reaching the IR temperature sensor system, enabling precise calculations of its values. In Test-1, RMSE is 1.183 °C; in Test-2, RMSE is 1.074°; and in Test-3, RMSE is 1.315 °C. During these tests, the maximal measurement error of the cooking vessel temperature was approximately ±3 °C. This underscores the functionality of the model, validating its accuracy.

**Figure 23:**
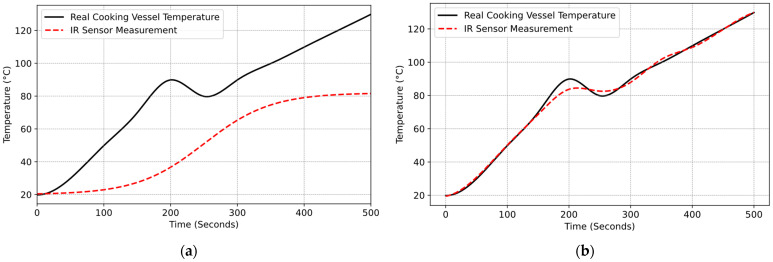
Real cooking experiments: (**a**) without optical filter (Pot A and ε  = 0.29); (**b**) with optical filter (Pot A and ε = 0.29).

After validating the model, real-time cooking experiments were conducted to validate the model’s ability to estimate cooking vessel temperature with and without the optical filter. To clearly understand the improvement in measurement, around the 200th second, cold water was added to the cooking pot to reduce its temperature. However, the IR sensor measurement did not reflect any cooling effect. Additionally, it was observed that the temperature of the cooking vessel could not be measured accurately because of the glass ceramic (Figure 23a). Afterward, the experiment was repeated with the optical filter integrated into the sensor system. Figure 23b shows that after adding the optical filter, it became possible to measure the temperature of cooking vessel and the cooling effect on the pot.

Before applying the optical filter, cooking vessel temperature could not be measured accurately. However, the maximal measurement error of the cooking vessel temperature was significantly reduced with the implementation of the optical filter, and the maximal measurement error was approximately ±3 °C. This improvement demonstrates the filter’s effectiveness in mitigating thermal radiation interference from the glass ceramic.

The ability to measure cooking vessel temperature in real time in induction hobs offers numerous benefits, with one of the most significant being an improvement in energy efficiency. In traditional power-controlled hobs, energy is continuously applied to the cooking vessel based on user-defined power levels, often resulting in overconsumption of energy. In contrast, our sensor-based system ensures more accurate control of the cooking vessel temperature, reducing unnecessary energy expenditure.

To quantify the energy efficiency of the proposed sensor-based system, we compared the energy consumed by traditional induction hobs (power-controlled) with that of our temperature-controlled system. We conducted a series of cooking experiments to measure the energy consumption of both the traditional and temperature-controlled induction hob systems. Each experiment involved maintaining a target cooking temperature (e.g., 200 °C) for a specific time period (e.g., 45 min) and measuring the energy consumed by each system. With the traditional hob, the power level was manually set to achieve the desired temperature, with no feedback control. With the temperature-controlled hob, the temperature sensor feedback was used to adjust the power in real time to maintain the desired cooking temperature. During the test, energy consumption was recorded using the Chroma Power Analyzer [29] device. Table 6 summarizes the experimental results for energy consumption during the cooking process.

The energy savings achieved by the sensor system are calculated as follows in Equation (14):

(14) $$∆E=E_{Trad}-E_{Sensor}$$

where $E_{Trad}$ is the total energy consumed by the traditional hob, $E_{Sensor}$ is the energy consumed by the temperature-controlled hob, and ∆E is the energy savings achieved by the sensor system.

The percentage improvement in energy efficiency (η) is then Equation (15):

(15) $$\eta =\frac{\Delta E}{E_{Trad}}*100\%$$

Across multiple tests, the temperature-controlled system consistently showed energy savings ranging from 28% to 33%. These savings are attributed to more precise temperature control, which minimizes overheating and the associated waste of energy.

In addition, incorporating the IR temperature sensor system into the design of induction hobs for temperature measurement and control introduces additional manufacturing costs. Proportionally, adding an external filter to the system increases the sensor system cost by 15%, which is considered quite reasonable in terms of practical applicability. Also, typically, the integration of a thermopile sensor for measuring from 120 °C entails higher expenses due to the requirement for custom window materials. These thermopile sensors, customized for induction hobs, are relatively costly—approximately 70% more expensive—compared to standard thermopile sensors, largely due to limited production volumes. In this solution, we used a separated optical filter that is inexpensive due to the availability of advanced, cost-effective materials and manufacturing processes. Despite the small cost increase with the optical filter, only regarding the sensor system around 15%, the optical filter significantly enhances temperature measurement accuracy. As a result, these manageable cost increases offer substantial long-term benefits in terms of improved energy efficiency and cooking performance.

## 4. Conclusions

This study presents a novel approach to IR temperature measurement in induction hobs by incorporating an optical filter to mitigate the interference from the heated glass ceramic. An optical filter designed to match the optical properties of glass ceramic offers excellent thermal stability, infrared transparency, and durability at high temperatures. Both simulations and real cooking experiments validate the effectiveness of the proposed system, demonstrating significant improvements in measurement accuracy and temperature control. The experimental results demonstrate that the inclusion of the optical filter reduced the maximal measurement error of cooking vessel temperature to approximately ±3 °C. This improvement enables precise temperature control, even at lower cooking temperatures, thereby optimizing cooking performance and reducing energy consumption.

Energy efficiency benefits stem from the system’s ability to maintain precise temperature control, minimizing wasted energy due to over- or underheating. Simulations and experimental data indicate a reduction in energy usage by approximately 30% compared to traditional systems, as the system avoids unnecessary energy dissipation caused by measurement inaccuracies.

The proposed system provides a cost-effective solution without compromising accuracy or durability. Incorporating the IR temperature sensor system with an external optical filter introduces a manageable 15% increase in sensor system costs, which is considered reasonable for practical applications. Unlike customized thermopile sensors with specialized window materials that are approximately 70% more expensive than standard sensors, the proposed solution utilizes a cost-effective separated optical filter.

These advancements have the potential to enhance cooking experiences, improve energy efficiency, and increase safety in domestic induction appliances.

While the current study successfully demonstrates the effectiveness of the proposed sensor system, future research should investigate the integration of advanced materials and coatings for optical filters, as well as exploring alternative sensor configurations for possible improvements in measurement accuracy and reliability.

## Figures and Tables

**Table 1:** Comparison of power level-controlled and temperature-controlled IH systems [9].

Power-Controlled Systems	Temperature-Controlled Systems
Only the power level can be selected by the user, and the actual cooking vessel temperature is unknown.	Target temperature values can be selected directly, and the hob alerts the user regarding actual temperatures.
Cooking outcomes heavily rely on the user’s skills and habits.	Repeatable and professional recipes are easy to follow for excellent cooking results.
There is a risk of smoke or fire if the power level is set too high or the cooking vessel is left unattended. This poses serious safety risks.	Hazardous situations are mitigated, as the cooking vessel temperature is continuously monitored and maintained.

**Table 2:** DoE list for thermopile selection.

No. of Experiment	No. of Sensor	Cooking Vessel	Power (W)	Temperature (°C)
1	1	A	2300	25–100
4	1	A	3400	25–250
28	4	A	3400	25–250
…	…	…	…	…
64	4	B	3400	25–250

**Table 3:** Specification of the thermopile temperature sensor.

Parameter	Specification
Sensor type	Single thermopile element
Communication	Digital (SMBUS)
Outputs	Object temp., self temp.
Window material	Optional (uncoated silicon, BPF)
Transmittance	≥85%, 3000–5000 nm (for BPF)
Emissivity of window	0.08 3000–5000 nm (for BPF)
Reflectance of window	0.07 3000–5000 nm (for BPF)
Time constant	Max. 20 msec
Accuracy	±1 °C
Responsivity	30 V/W (without filter)
Specific detectivity	0.6 10^8^ cm√Hz/W
Operating temperature	(−20 °C) … (+100 °C)
Measurement range	(−20 °C) … (+400 °C)
Field of view	20°
FoV tolerance	X-axis: ±2°, Y-axis: ±2°
Supply voltage	5 V (±0.5 V)
Noise voltage for thermopile	25 nV/√Hz/W
Current consumption	Max 7 mA
Output current	Max 80 mA

**Table 4:** Simulation parameter set.

	Transmittance (τ)	Emissivity (ε)	Reflectance (ρ)
Cooking vessel	0	0.59	0.41
IR sensor window	≥85%, 3–5 μm (BPF)	0.08%, 3–5 μm (BPF)	0.07%, 3–5 μm (BPF)
Glass ceramic	$\tau_{C}$	$0.95-\tau_{C}$	0.05

**Table 5:** Simulation parameter set with optical filter.

	Transmittance (τ)	Emissivity (ε)	Reflectance (ρ)
Cooking vessel	0	0.59	0.41
Optical filter	$\tau_{C}$	$0.95-\tau_{C}$	0.05
IR sensor window	≥85%, 3–5 μm (BPF)	0.08%, 3–5 μm (BPF)	0.07%, 3–5 μm (BPF)
Glass ceramic	$\tau_{C}$	$0.95-\tau_{C}$	0.05

**Table 6:** Experimental results for energy consumption.

No. of Test	Target Temperature (°C)	Time (Minutes)	Traditional Energy (Wh)	Temperature-Controlled Energy (Wh)
1	100	30	568	381
2	150	45	1314	920
3	200	45	1861	1340

## Data Availability

Data are contained within the article.
